# Cohesiveness of Tin Fillings

**Published:** 1894-07

**Authors:** Charles E. Francis

**Affiliations:** New York


### COHESIVENESS OF TIN FILLINGS.[1]

BY DR. CHARLES E. FRANCIS, NEW YORK.

In a paper which I read at the last meeting of this Society, I referred to tin as a valuable material for filling a certain class of cavities in teeth which my paper described, and also remarked that refined tin, or tin in a state of absolute purity, possessed a decided cohesive property. For making this statement, I was gently corrected by several friends present, who asserted that tin, or preparations of this metal, to which my paper referred, possessed no cohesive tendencies whatever. Although it was shown by specimens presented, in evidence of what I stated, that large contour fillings could be produced with pure tin-foil or tin shavings, which were so thoroughly condensed as to be firmly held together, and sufficiently compact as to receive smooth and brilliant surfaces, it was, nevertheless, declared by those who differed with me in opinion, that no actual union of the layers of this metal existed as they were forced against each other, but that they were held together simply by an intermingling of metal fibres produced by sharp points, or serrations.

In using the term "cohesion" on the occasion referred to, I presumed that I was correct in so doing; but as my nomenclature was questioned, I determined to look up authorities on the subject, with a view of either substantiating the correctness of my statement or admitting that I was in error.

My idea of cohesion (as before expressed) is the attractive force which holds or binds together the atoms, particles, or molecules of any homogeneous mass. In solids, it is the strength or integrity of the substance, and it matters not how or in what manner the atoms are blended.

Cohesion of metals may be effected by heat or pressure, according to the nature of the material. In the use of tin-foil, or shavings, where the layers are massed together to form a compact filling in a tooth, these layers are united or held together by the law of cohesion; and it matters not whether they adhere or stick together by slight pressure, like strips of damp court-plaster, or that their fibres are intermingled, interwrought, or blended together by means of serrated or wedge-shaped instruments. So long as the layers of foil are held together in such a manner as to resist any degree of tension, they are united by cohesive attraction; and it is not neces-

[1] Read before the New York Odontological Society, April 17, 1894.

sary that the mass of metal should be absolutely *solid*, according to the general acceptation of this term, to define the attractive force as "cohesion;" for everybody is supposed to know that all substances which are denominated solids are but bodies of molecules held together by this force.

In regard to the tin fillings which I have produced, and some of which have been here exhibited, it is evident that the particles of metal are quite firmly united, and capable of resisting a considerable degree of strain. According to my idea of the term "cohesion," these particles, or layers, are bound or held together by this force; and I believe that I am supported in this view by popular authorities.

[Dr. Francis then quoted from various encyclopædias and popular text-books definitions of the term "cohesion;" also read several letters from prominent professional gentlemen to whom he had written for opinions concerning the cohesive properties of tin; then concluded by stating that if he was in error in denominating as "cohesive attraction" the force which holds together the atoms of tin in fillings of this material, that he was not alone in such opinion.]

